# Salvage Therapy with Rezafungin for *Candida parapsilosis* Spondylodiscitis: A Case Report from Expanded Access Program

**DOI:** 10.3390/microorganisms12050903

**Published:** 2024-04-30

**Authors:** Giulio Viceconte, Antonio Riccardo Buonomo, Nunzia Esposito, Letizia Cattaneo, Teresa Somma, Maria Michela Scirocco, Ciro Gabriele Mainolfi, Ivan Gentile

**Affiliations:** 1 Department of Clinical Medicine and Surgery, Section of Infectious Diseases, University of Naples “Federico II”, 80131 Naples, Italy; antonioriccardobuonomo@gmail.com (A.R.B.); espositonunzia.94@gmail.com (N.E.); letizia.cattaneo90@gmail.com (L.C.); mmichelascirocco@hotmail.com (M.M.S.); ivan.gentile@unina.it (I.G.); 2 Department of Neurological Sciences, Division of Neurosurgery, University of Naples “Federico II”, 80131 Naples, Italy; teresa.somma85@gmail.com; 3 Department of Advanced Biomedical Sciences, University of Naples “Federico II”, 80131 Naples, Italy; cirogabriele.mainolfi@unina.it

**Keywords:** rezafungin, *Candida*, spondylodiscitis, osteomyelitis

## Abstract

*Candida* spp. spondylodiscitis is a rare condition for which treatment options are often limited. A further obstacle is the duration of therapy, which should be administered for up to twelve months. In view of the long duration of therapy, azoles are, so far, the only oral treatment strategy that can be given as home therapy. In the case of resistance or reduced susceptibility to azoles, there are not enough comfortable treatment opportunities with adequate bone penetration and limited toxicity. We report the first case of the successful use of rezafungin for spondylodiscitis due to *Candida parapsilosis* with reduced susceptibility to azoles. A 68-year-old patient, affected by paraplegia and short bowel syndrome, was diagnosed with *Candida parapsilosis* spondylodiscitis, confirmed with a culture on vertebral biopsy after an 18-FDG PET/CT scan. He received 200 mg of rezafungin weekly for 26 weeks, after 10 weeks of previous antifungal treatment that was not well tolerated with voriconazole plus liposomal amphotericin B. He had a full clinical, radiologic, and biochemical response to the therapy with rezafungin, with no adverse effects. Rezafungin can be a promising therapy for Candida osteomyelitis, especially when first line therapies are ineffective, poorly tolerated, or contraindicated.

## 1. Background

Spondylodiscitis are infections that affect the spine, particularly the vertebrae and intervertebral discs. Spinal infections can be described etiologically as pyogenic, granulomatous (tuberculous, brucellar, fungal), and parasitic [1]. Spreading to the vertebrae and intervertebral discs can occur either by hematogenous seeding or by exogenous inoculation [1].

Invasive candidiasis is the infection of a sterile site by *Candida* spp. and includes both candidemia and deep-seated tissue candidiasis, which arises from the dissemination of *Candida* spp. to a sterile body site (e.g., endocarditis, peritonitis, endophthalmitis) [2].

*Candida* spp. spondylodiscitis (CS) is a rare manifestation of invasive candidiasis; only 89 cases of culture-confirmed CS have been reported so far, according to a recent meta-analysis [3]. *Candida albicans* spondylodiscitis was the most frequent species isolated and only six cases of *C. parapsilosis* were described [3].

*C. parapsilosis* represents a high risk for immunocompromised individuals and surgical patients, particularly those subjected to gastrointestinal track surgery. The incidence of *C. parapsilosis* infections in Europe is region-dependent; in Southern European hospitals (Portugal, Spain, Italy, and Greece), it is the second most isolated species [4].

The latest results from the European Confederation of Medical Mycology (ECMM) Candida III study, an observational study assessing *Candida* spp. distribution and the antifungal resistance of candidemia across Europe, found that acquired fluconazole resistance was common in *C. glabrata* and *C. parapsilosis* and had a 24% rate of fluconazole resistant *C. parapsilosis* in Greece, Italy, and particularly Turkey [5].

According to a meta-analysis by Adelhoefer and colleagues, among the 89 included patients with CS, antifungal monotherapy was given in 58% of cases; the most commonly used antifungal agents were fluconazole (68%), amphotericin B (38%), and echinocandins (26%), and the median length of antifungal treatment was six months [3]. Surgical intervention was performed in 68% of cases, including 34% undergoing instrumented discectomy [3]. At a median follow-up of 12 months, 3% developed sepsis, 6% underwent revision, and 12% died of disease [3]. Younger age (*p* = 0.042) and longer length of antifungal therapy (*p* = 0.061) were predictive of survival, whereas the outcome did not differ based on Candida strain (*p* = 0.74) and affected spinal level (*p* = 0.44) [3].

No definitive indications for the treatment of CS exist, but the Infectious Diseases Society of America (IDSA) recommends treating *Candida* spp. osteomyelitis with 6 mg/kg daily of fluconazole for 6 to 12 months, or an echinocandin or liposomal amphotericin B (L-AmB) for 2 weeks, followed by long-term fluconazole [6].

Rezafungin is a novel, semisynthetic, long-acting, second-generation echinocandin derived from anidulafungin with favorable pharmacokinetic properties due to structural modifications [7]. It has several advantages over the already approved echinocandins as it has better tissue penetration, better pharmacokinetic/pharmacodynamic (PK/PD) pharmacometrics, and a good safety profile [7]. Rezafungin exhibits superior stability in solution compared to older echinocandins, enhancing its versatility in dosing, storage, and production [7]. This enhanced stability facilitates once-weekly administration via intravenous route and holds potential for topical and subcutaneous application. Furthermore, higher dosage regimens have been evaluated without any observed toxic effects, which could ultimately mitigate the emergence or proliferation of resistant strains [7].

Despite *C. parapsilosis* isolates having shown the highest in vitro MICs for rezafungin (geometric MIC 1.657; MIC range 0.063–>4 mg/L) among all the Candida species, according to a multicenter study across four European laboratory [8], rezafungin was able to inhibit 100% of *C. parapsilosis* isolates at ≤4 μg/mL with MIC50/90 of ½ μg/mL in a worldwide collection of 2205 invasive fungal isolates recovered from 2016 to 2018 and interpretated with Clinical and Laboratory Standards Institute (CLSI) broth microdilution methods [9].

In the phase 2 (STRIVE) trial, adults with candidemia and/or invasive candidiasis were randomized to receive either 400 mg of rezafungin once weekly or 400 mg of rezafungin in week 1 followed by 200 mg once weekly or 70 mg of caspofungin as a loading dose, followed by 50 mg daily for ≤ 4 weeks [10]. The overall cure rate was highest for 400/200 mg of rezafungin compared to 400 mg of rezafungin or caspofungin (76.1% vs. 60.5% vs. 67.2%, respectively) and the mortality rate was lowest for 400/200 mg of rezafungin compared to 400 mg of rezafungin or caspofungin (4.4% vs. 15.8% vs. 13.1%, respectively), with candidemia clearing earlier in patients on rezafungin than those receiving caspofungin, although the trial was not powered for inferential analysis [10].

Moreover, in the phase 3 (ReSTORE) trial, adults with systemic signs and mycological confirmation of candidemia or invasive candidiasis were randomly assigned (1:1) to receive intravenous rezafungin once a week (400 mg in week 1, followed by 200 mg weekly, for a total of two to four doses) or intravenous caspofungin (70 mg loading dose on day 1, followed by 50 mg daily) for no more than 4 weeks [11]. The global cure rate at day 14 was 59% in the rezafungin group, compared to 61% in the caspofungin group, while 30 day mortality was 24% and 21% in the rezafungin and caspofungin groups, respectively, thus showing non-inferiority of rezafungin to caspofungin [11].

Nonetheless, patients with osteoarticular infections were excluded from the abovementioned trials and only a few case reports have described its use for more than 4 weeks and for infections outside the bloodstream [12,13,14].

We hereby report the first use of rezafungin for a CS due to *Candida parapsilosis* with reduced susceptibility to azoles.

## 2. Case Report

A 68-year-old patient was admitted to our ward for persistent febrile episodes. The patient’s remote history was characterized by the following: paraplegia due to vertebral trauma after a car accident, with complete injury at the D4–D6 level, stabilized with trans-peduncular bars and screws and complicated with sub lesional syringomyelia and complete sub lesional anesthesia; short bowel syndrome after ileo-cecal resection with latero-lateral ileo-colic anastomosis, due to bowel volvulus, on home parenteral nutrition via peripherally inserted central venous catheter (PICC); paroxysmal atrial fibrillation; and a neurogenic bladder on self-catheterization.

The patient reported the onset of fever and subcutaneous swelling at the D7–D9 level from March 2023. Blood cultures collected at home from the peripheral vein in May 2023 yielded azole resistant *Candida parapsilosis*, for which he had not received therapy. A magnetic resonance imaging (MRI) examination in May 2023 showed fluid collection at the level of the subcutaneous tissue at the D7–D8 and L4–L5 spondylodiscitis. The patient was then admitted to the Infectious Diseases Ward of Federico II University Hospital at the beginning of June 2023.

Blood cultures and the tip of the removed PICC yielded *C. parapsilosis* resistant to posaconazole, voriconazole and fluconazole (Table 1). The therapy with anidulafungin 100 mg i.v. daily was started after a 200 mg loading dose for the treatment of candidemia, with negative surveillance blood cultures at 72 h. The patient underwent transesophageal echocardiography and fundus oculi examination, as recommended for candidemia, and both were negative for metastatic localization.

**Figure 1:**
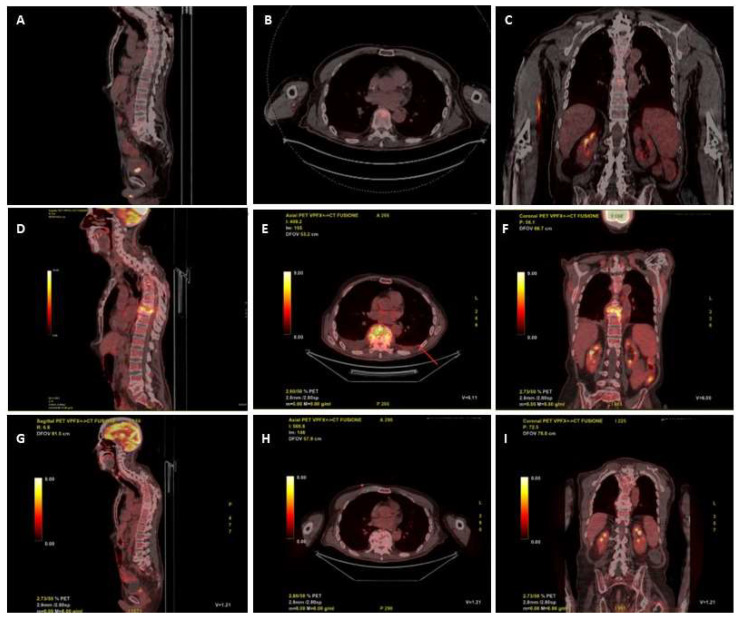
Digital PET/CT scan reconstruction (Discover GE) after administration of 250 mCi of 18-FDG: sagittal (**A**), axial (**B**) and coronal (**C**) planes at diagnosis of spondylodiscitis; sagittal (**D**), axial (**E**) and coronal (**F**) planes after 11 weeks of rezafungin: sagittal (**G**), axial (**H**) and coronal (**I**) planes after 26 weeks of rezafungin.

An 18-FDG PET/CT scan requested on admission for suspected vertebral infection level showed a focal increased uptake in the body of D9 (SUV max 9.5) and in L4–L5 (SUV max 5.9) (Figure 1A–C). Ten days after the start of the anidulafungin therapy, the patient underwent a trans-peduncular bone biopsy of D9, on which the culture yielded fluconazole resistant *C. parapsilosis*, with increased exposure sensitivity to voriconazole and sensitivity to itraconazole and posaconazole (Table 1). The histopathologic examination of the biopsy was unremarkable, mostly due to the scarcity of the sample.

In order to facilitate the patient’s discharge to the outpatient parenteral hospital therapy service, antifungal therapy was switched to 4 mg/kg of voriconazole orally every 12 h plus 10 mg/kg i.v. of liposomal amphotericin B three times a week. He started this therapy while he was still hospitalized. Meanwhile, our center applied for a rezafungin expanded access program. The rationale for this decision lies in several factors: the differences in susceptibility profiles of *Candida parapsilosis* in blood and in bone, the length of antifungal therapy required for the treatment of Candida spp. osteomyelitis, and the concern about the actual absorption of an oral therapy because of the short bowel syndrome from which the patient suffered.

Seven days after the start of the therapy with voriconazole plus liposomal amphotericin B three times a week, the patient developed insomnia, hallucinations, prolonged QTc interval, and hypokalemia as side effects of the current antifungal therapy. For this reason, on 16 August, antifungal therapy was stopped, the patient was discharged at home, and he received a loading dose of 400 mg i.v. of rezafungin, followed by 200 mg weekly.

He remained in good conditions, with persistently negative serum beta-D-glucan (BDG), through all the follow-up, during which he developed, in September and in October, two PICC-related bloodstream infections due to methicillin-resistant *Staphylococcus haemolyticus*, both treated with a 1500 mg i.v. of dalbavancin and catheter removal. A new 18-FDG PET/CT was requested at the end of October, after 11 weeks of rezafungin, which showed an increased glucose uptake between the D9 and D10 (SUV 15.6), with no uptake at the L4–L5 level (Figure 1D–F). A subsequent contrast enhanced spine MRI, performed two days later, showed signs of D8–D9 spondylodiscitis with epidural abscess between D7 and D11. Despite the patient being stable, afebrile, and with negative C-reactive protein (CRP) and BDG, on the suspicion of therapeutic failure or coexistence of other diseases (neoplasm, mycobacterial, or staphylococcal superinfection), other multiple vertebral biopsies were collected at D8 and D9, all resulting in negative for mycobacteria (molecular, acid fast stain and culture), fungi, and bacteria, and with non-specific histopathology. Despite the unexplained radiological worsening, rezafungin was continued.

The patient remained in good conditions, with persistently negative serum BDG and CRP during follow-up. A new 18-FDG PET/CT performed after 36 weeks of the antifungal treatment (and after 26 weeks of rezafungin) showed remarkable reduction in the 18-FDG uptake (SUV 2 vs. 15.6) at the D9–D10 and no pathologic uptake at the L4–L5 (Figure 1G–I); thus, antifungal therapy was stopped.

In summary, the patient received 200 mg weekly of rezafungin for 26 weeks (and a total of 36 weeks of effective antifungal therapy, when considering the previous therapies). He is still in good clinical condition, with negative CRP and serum BDG.

## 3. Discussion

To the best of our knowledge, this is the first report of successful use of rezafungin for the treatment of an osteoarticular infection due to *Candida* spp., and one of the few cases in which it has been used for more than 4 weeks. In fact, in both phase 2 and 3 trials, the longest administration of rezafungin permitted per protocol was 28 days [10,11]. Moreover, patients with *Candida*-associated septic arthritis in a prosthetic joint, osteomyelitis, endocarditis, or myocarditis, meningitis, chorioretinitis, CNS infection, as well as those affected by chronic disseminated candidiasis, or urinary tract candidiasis, were excluded from the trials [10,11].

Compassionate use of rezafungin for difficult-to-treat Candida infection has been reported in one case of aortic graft mediastinal infection, in which it has been used for more than 1 year, one case of chronic mucocutaneous candidiasis in a patient with primary immunodeficiency (5 weeks), and one case of intrabdominal candidiasis in a liver transplant recipient (12 weeks) [12,13,14].

Despite the fact that we could not find any study reporting rezafungin pharmacokinetics data in bone tissue, the rationale for using rezafungin in fluconazole-resistant *Candida* spp. osteoarticular infections relies on the bone penetration of anidulafungin, which, based on animal models, reaches values similar to plasma concentrations, regardless of dosing duration, with a bone/plasma concentration ratio of approximately 1.0 in neonatal rats [15].

Other antifungal agents have been proven to reach a bone/plasma concentration ratio ranging from > 0.5 to ≤ 5 (itraconazole and 5-flucystosin) or > 5 (voriconazole, amphotericin B deoxycholate) [16]. Nonetheless, these drugs are characterized by high toxicity, needing therapeutic drug monitoring or intravenous daily dosing, which makes them not appealing for a prolonged therapy. Conversely, rezafungin has shown to be safe, with no need for dose adjustment by age, body surface area, and albumin levels in pharmacokinetic models [17], with similar outcomes in obese patients in the phase 2 trial [18] and absence of significant drug–drug interactions [19].

To overcome some of the limitations of the current guidelines on candidemia and invasive candidiasis, the ECMM, in cooperation with the International Society for Human and Animal Mycology (ISHAM), has recently developed an initiative called the New Global Guideline for the Diagnosis and Management of Candidiasis, which has recently undergone a public review and has still to be published. It is expected that rezafungin will receive a first-line indication for the treatment of candidemia alongside echinocandin, but the role of this new molecule for the treatment of deep-seated infections is still to be clarified.

In our experience, the coexistence of *quasispecies* of *Candida parapsilosis* in blood and bone, including some azole-resistant strains, together with the presence of a short bowel that might have impaired adequate therapeutic level of voriconazole, the development of toxicity from voriconazole and amphotericin, as well as the need for a long-term treatment, made our patient the ideal candidate for rezafungin via the expanded access program.

Our patient had a satisfactory clinical and biochemical response to rezafungin, with no adverse reaction reported during therapy. We still cannot fully explain the increased FDG uptake that we found at the intermediate follow-up 18-FDG PET/CT. We speculate that the patient underwent the first PET too early in the history of the infection; thus, we cannot exclude that the radiological picture we found in October 2023 reflected an alteration that occurred in the first week after the first PET was performed. Also, because the patient has complete sub lesional anesthesia below D4, we could not rely on symptoms of pain or neurological worsening.

## 4. Conclusions

Rezafungin seems to be a promising therapeutic option for patients with *Candida* osteomyelitis and with difficult-to-treat IC in general, especially when oral azole therapy is not an option. Further studies are required to understand the exact duration of therapy, the right dose, the need for therapeutic drug monitoring, and the need for combination with other antifungals.

## Figures and Tables

**Table 1:** Antifungal susceptibility profiles of clinical isolate from blood cultures and bone. Interpretation according to the Clinical and Laboratory Standards Institute (CLSI); posaconazole and amphotericin B according to European Committee Antimicrobial Susceptibility Test (EUCAST).

	Blood Cultures		Bone	
	*Candida parapsilosis*		*Candida parapsilosis*	
DRUG	MIC (mg/L)	Interpretation	MIC (mg/L)	Interpretation
Caspofungin	1	S	0.5	S
Fluconazole	128	R	32	R
Voriconazole	1	R	0.5	I
Anidulafungin	1	S	1	S
Micafungin	2	S	2	S
5-flucytosine	0.12	S	0.06	S
Posaconazole	1	R	0.06	S
Itraconazole	0.25	I	0.12	S
Amphotericin B	0.5	S	1	S

## Data Availability

Data are contained within the article.
